# Experimental Physiology

**Published:** 1870-09

**Authors:** J. L. Prevost

**Affiliations:** Member of the Biological Society


### Original Translations.

ARTICLE I.—*Experimental Physiology.* Experimental Investigations relative to the Directions of Rotary Movements due to Unilateral Encephalic Lesions. By Dr. J. L. PREVOST, Member of the Biological Society.

[ Continued from the August Number, 1870.]

The two ocular globes being turned habitually from the right side, the two sclerotics being apparent from the left side of the animal, although the dog could carry his two eyes to the left but with less power than to the right, there were slight nystagmic movements in the two eyes.

The right pupil was more dilated than the left, but they were both contractile under the influence of light, and they were both equally dilated by a solution of atropine.

When food was presented to the animal and placed at his left he did not see it; when, on the contrary, placed at his right he saw it readily and seized it. I, at first, suspected hemiopia; but after repeated examination of both eyes, became convinced that he saw well with the right eye, and that vision was, on the contrary, abolished in the left. Indeed, by closing the right eye of the animal and threatening the left with the finger, no reflex movements were induced so long as neither the cornea nor the lid were touched. The same experiment tried upon the right side resulted differently, and the dog winked at the slightest movement of the finger which threatened him. By an ophthalmoscopic examination I discovered no lesion of the left eye, the appearance of the bottom of the eye was similar on both sides. The dog has continued a rotary movement from left to right; but he does not execute it constantly, when he wishes to walk, as in the first days. When the animal has a strong desire to direct his steps toward an object, as when food is placed at a certain distance from him, he advances straight toward it, when, without any apparent cause for the phenomenon, he executes a rotary movement from left to right, sometimes several, resumes his direct advance, then frequently again performs the same evolution once or twice before reaching the object; sometimes, on the contrary, he reaches it directly.

When the animal is left to himself in the apartment, and walks without any direct object, he turns almost constantly, spirally, (en manége) from left to right. When he eats, and when he is placed before the vessel which contains his food, he executes from time to time spiral turns, always in the same direction.

When a morsel of food is placed very near the left side of his muzzle, in place of turning his muzzle slightly to the left to seize the prey, he prefers to execute a spiral movement to seize the food as he meets it in the course of the movement. This tendency to rotary movement to the right is very manifest when by causing the dog to follow one, he is made to pass circularly around the room. When the circuit or grand spiral is made from left to right the animal has no difficulty in following it, however he finds himself compelled to execute from time to time what horsemen would term a turn to the right, then he continues upon the grand spiral. It is only with the greatest difficulty that the animal traverses a grêat circle from right to left, around the apartment; he increases the frequency of his turns, or springs to the right, and he can rarely accomplish the circuit of the apartment.

This involuntary impulsion into the rotary movement, from left to right, becomes still more remarkable when the animal attempts to go up or down stairs; he advances at first directly, ascends several steps, then hesitates, apparently resisting an invincible force which compels him to execute his spring to the right; he sometimes resists but often yields; he acts then cautiously, in order not to roll down stairs, which sometimes happens to him; often he groans and manifests his impatience at being obliged to yield to the impulse which forces him to turn.

It is not the loss of the left eye which directs the animal; for the same phenomena are produced when the right eye is closed, or even when a handkerchief is placed covering the two eyes.

These symptoms continued up to the day when the animal was killed; however, during the latter part of the time the tendency to rotation seemed to have slightly diminished, and the dog in advancing directly executed the spiral movements less frequently.

I had the opportunity to observe this dog for a long time, and to determine on several occasions the interesting phenomena which

I have deemed it my duty to give in detail, as possessing much importance, in regard to the theory of rotary movements. I have demonstrated them to the members of the Biological Society, before which I exhibited this dog at two different epochs, before and after his complete cure, and I moreover exhibited to the Biological Society the cerebral lesions found after his death.

This dog was killed the 18th of January, 1867.

*Necropsy.* Nothing in the meninges, except a slight circumscribed adhesion of the dura-mater to the right hemisphere at the seat of the wound of the brain.

*Right hemisphere.* A little triangular wound was observed situated upon the sphenoidal lobe, at the anterior margin of the fourth convolution, counting from the interhemispheric fissure, and upon the portion of this convolution which extends anteriorly beyond the third convolution, which is shorter than the fourth. In penetrating deeply, the instrument passed the inferior portion of the right corpus striatum, leaving the optic thalamus on its inner side. It traversed in its external portion the right optic tract, of which about half the thickness was lacerated. Then, penetrating obliquely into the superior portion of the right cerebral peduncle, the gimlet traversed this peduncle step by step, and escaping at the base of the brain, a little below the tubercula mammillaria, was stopped probably by the bone, which bore no trace of injury.

The point of emergence of the instrument forms a little rounded wound about one millimetre in diameter, and distant from the adjacent parts as below:

| | | |
|---|---|---|
| Distance from the inferior portion of the mammillary tubercles, - - - - - - - | 1½ | millimeters. |
| Distance from the inter-peduncular line, - - | 3 | " |
| " " " external line of the right peduncle, - | 8 | " |
| " " " superior portion of the protuberance, | 8 | " |

The trace of the wound is more or less anfractuous, its walls are reddish, slightly yellowish in portions, and are formed of cerebral substance mixed with blood and degenerated.

Microscopic examination detected in these portions of the debris, nervous elements, a large number of granular bodies, some of which are formed by simple agglomeration of fatty granulations, whilst others present an envelope, a nucleus, and a nucleolus.* There is, moreover, a very rich proliferation of nuclei of vessels, and an incipient formation of an interstitial cellular network, and a very few globules of hæmatosine.

The left hemisphere, the tubercula quadrigemina, and the other portions of the brain, were healthy.

The two eyes were examined, and presented no alteration.

It was not possible to determine, either with the naked eye or by microscopic examination, degeneration of the right anterior pyramid, nor of the opposite half of the cord.

WOUND OF THE RIGHT HEMISPHERE, EXTENDING TO THE EXTERNAL PORTION OF THE CORPUS STRIATUM; FORMATION OF AN ABSCESS IN THIS COURSE; ROTATION OF THE HEAD AND OF THE EYES TO THE RIGHT; VERY MARKED REVOLUTION FROM THE LEFT TO THE RIGHT.

*Exp.* II. Small adult bitch, of the terrier breed.

On the 6th of December, 1867, I perforated with a gimlet, having a diameter of two to three millimetres, the cranial vault of the right side, and buried the instrument to a certain depth in the cerebral substance: no symptom followed.

I effected a new perforation at a little distance from the first, and I buried still more deeply the instrument in the right cerebral hemisphere.

Immediately the animal commenced to turn from left to right, a spiral movement with a short radius. The head was slightly inflected upon the axis of the neck, the vertex being directed from the left side of the animal, and the left eye being upon a lower plane than the right; the muzzle, on the contrary, was turned to the right shoulder.

The two ocular globes were directed both strongly from the right side, the left iris touching the internal angle, the right iris the external angle of the palpebral openings. The sclerotic of the left eye was apparent in the external angle, and that of the right eye in the internal angle.

* M. Bouchardat has very well described these two kinds of granular bodies, attributing the first to *necrobiosis*, the second to inflammatory action. (See Thèse de M. Poumeau, p. 110 et suiv. Paris, 1869).

Upon elevating the head, which was easily done, for there appeared to be no stiffness of the neck, the deviation of the ocular globes from the right side appeared yet more marked.

In the act of turning, the animal threw himself frequently against objects which he met, and appeared to see imperfectly.

There was also perceptible a slight hemiplegia of the left side, especially in the anterior paw. The animal, when pushed, appeared to fall more readily upon the left side than upon the right.

12th of December. The same symptoms have persisted, and to-day they are still more decided. The animal suffers and groans. When placed upon his feet he is exhausted, he bends himself into an arc of a circle, with the concavity turned to the right. The muzzle is directed to the right, and approximates nearly to the right thigh. The two ocular globes are both turned from the right side. In walking, the animal describes a spiral movement from left to right, forming a curve with a radius smaller even than upon the day of the operation.

The animal was killed by hanging.

*Necropsy.—Right hemisphere.* The cerebral meninges exhibit nothing particular. Two small wounds are observed upon the occipital vault; one very superficial, extending scarcely through the gray substance, and evidently produced by the first puncture which I made.

The second wound, situated more externally, penetrates deeply, and after having traversed the white substance of the centrum ovale, reaches the external portion of the corpus striatum. At this point, a large purulent focus has been formed, which is located especially at the level of base of the corpus striatum and the peduncular irradiation. This collection (of pus) attains a diameter about equal to that of the cerebral peduncle.

The pus has penetrated into the right ventricle, and thence has spread into the third, thence by way of the calamus scriptorius, it has been diffused over the superior portion of the cord, which is enveloped in pus and false membranes.

In this purulent collection are many granular leucocytes and true non-nucleated granular bodies, evidently formed by fatty granulations aggregated into groups. There is no appreciable degeneration of the peduncle, nor of the anterior pyramid.

The *left hemisphere* is healthy, as are also the cerebellum and its peduncles.

WOUND OF THE RIGHT HEMISPHERE; SLIGHT ROTATION FROM LEFT TO RIGHT; ROTATION OF THE HEAD AND EYES TO THE RIGHT.

*Exp.* III. Rabbit. January 10, 1868.

I perforated with a gimlet the vault of the cranium on the right side, and buried the instrument into the cerebral substance. There followed a rotary movement from the left to the right, which continued two or three minutes; the left pupil was contracted, the right dilated, the left eyelid depressed.

No perceptible hemiplegia, hebetude; after half an hour the pupils were equal, dilated, both lids being open. The animal has a tendency to turn the head from the right side, and when he is excited he turns more frequently to the right than to the left. Slight tendency to spiral movement from left to right.

*Necropsy.* Meningeal hæmorrhage, which surrounds the base of the brain and the medulla oblongata.

The injury was apparent upon the external portion of the *right hemisphere*, at the level of the union of the frontal with the sphenoidal lobe. The right olfactory peduncle was slightly lacerated. There was little blood in the ventricle. The deep portions (corpora striata, optic thalami, tubercula quadrigemina) were not involved.

WOUND OF THE RIGHT HEMISPHERE; NO SPIRAL MOVEMENT; SLIGHT ROTATION OF THE HEAD TO THE RIGHT.

*Exp.* IV. Rabbit. January 17, 1868.

By the same process I wounded the brain. There was no manifest spiral movement produced, but it was observed that the animal had a tendency to turn the head rather to the right than to the left, and when it was irritated it turned itself more frequently from the right side than from the left.

The pupils were equal; the eyes not sensibly deviated.

January 18. The preceding symptoms are no longer appreciable, and the rabbit turns as well from one side as from the other when frightened. He was killed.

*Necropsy.* Slight meningeal hæmorrhage at the base. The instrument had reached the anterior and external portion of the right frontal lobe; the cerebral substance was mingled with blood. The olfactory peduncle was cut throughout its whole thickness. The right corpus striatum was very slightly lacerated in its external and anterior portion.

WOUND OF THE RIGHT HEMISPHERE AND OF THE CORPUS STRIATUM; NO SPIRAL MOVEMENT; SLIGHT ROTATION OF THE HEAD TO THE RIGHT.

*Exp.* V. Rabbit. January 17, 1868.

The same operation. The rabbit described no spiral, but had a tendency to incline the head to the left, carrying the muzzle to the right; the eyes were slightly turned to the right (?). The left eye was more prominent, the pupil more dilated, than the right. Very slight nystagmus.

January 18. Nothing appreciable. The rabbit was killed.

*Necropsy.* The instrument had penetrated the right hemisphere in the plane of the union of the frontal with the sphenoidal lobe. The hemisphere was transfixed, and at its base; the wound was situated about two millimetres outside of the origin of the right olfactory peduncle.

The instrument had wounded the posterior-external portion of the corpus striatum, and slightly the anterior-external portion of the optic thalamus. A collection of blood extends a little below the optic thalamus into the anterior extremity of the sphenoidal lobe.

WOUND OF THE RIGHT HEMISPHERE; OPTIC THALAMUS; ROTATION OF THE HEAD AND OF THE EYES TO THE RIGHT; TENDENCY TO SPIRAL MOVEMENT FROM LEFT TO RIGHT.

*Exp.* VI. Rabbit. January 13, 1868.

I perforated the cranium and wounded the brain.

The head was inclined from the left side and in rotation to the right, the muzzle was directed from the side of the right shoulder. The eyes were slightly divergent to the right, which was especially apparent in elevating the head of the animal; the sclerotic then appeared from the side of the external of the left eye, and from that of the internal palpebral angle of the right eye.

When the animal was excited, there was observed a slight tendency to turn spirally, describing a circle of great radius.

*Necropsy.* The wound had extended to the level of the middle of the right hemisphere, had traversed the lobe, lacerated the tentorium of the right side, as well as the anterior portion of the right optic thalamus, which was infiltrated with blood.

The wound had not involved the deeper portions of the optic thalamus.

The corpus striatum and the other portions of the encephalon were uninjured.

WOUND OF THE RIGHT HEMISPHERE, CORPUS STRIATUM AND OPTIC THALAMUS; ROTATION OF THE HEAD AND OF THE EYES TO THE RIGHT; SPIRAL MOVEMENT FROM LEFT TO RIGHT.

*Exp.* VII. Rabbit. January 17, 1868. The same operation. The rabbit turned his head to the right, inflecting it slightly from the left side, in such manner as to direct the vertex slightly from the left side. The ears are both at the left of the animal. The eyes are both plainly turned to the right. The sclerotic appeared at the external angle of the left, and at the internal angle of the right eye. Left pupil more dilated than the right. Slight nystagmus.

When the animal is excited he executes a spiral movement, of short radius, from left to right.

The left fore paw seems to be a little less feeble than the right.

January 18. The same symptoms of very slight left hemiplegia and of rotation spirally from left to right. Pupils equal.

The rabbit was killed.

*Necropsy.* The instrument had wounded the upper portion of the right hemisphere in the plane of the union of the frontal with the sphenoidal lobe, at about two millimetres from the interhemispheric fissure. It had passed outside of the right corpus striatum, which was, as it were, raised and detached, and had wounded the right optic thalamus, of which the nervous substance was broken up and mingled with blood.

WOUND OF THE LEFT OPTIC THALAMUS; ROTATION OF THE HEAD AND OF THE EYES TO THE LEFT; SPIRAL MOVEMENT FROM RIGHT TO LEFT.

*Exp.* VIII. Rabbit. January 15, 1868. By the same process I wounded the brain.

The animal bent his head from the right side, turned it to the left, the muzzle being carried from the side of the left shoulder; the two eyes were slightly deviated to the left.

The animal, when excited, described a spiral movement from right to left with a large curve.

January 16. The same condition, the pupils equal, head and eyes turned to the left, spiral movement from right to left.

January 17. *Necropsy.* There was observed upon the upper surface of the right hemisphere in the plane of the median portion, in an antero-posterior direction, and about one millimetre from the interhemispheric fissure, a little wound about one millimetre in diameter.

In penetrating deeply, the instrument passing obliquely from the left side, traversed the corpus callosum in the median line and wounded the anterior wall of the third ventricle, which contained blood and brain substance mixed with blood. It was observed that the left wall of the third ventricle (optic thalamus) was broken up and infiltrated with blood, especially at the level of its anterior portion, whilst the right optic thalamus was simply very slightly scraped, and exhibited perfectly normal consistence and color.

WOUND OF THE LEFT OPTIC THALAMUS; ROTATION OF THE HEAD AND OF THE EYES TO THE LEFT; SPIRAL MOVEMENT FROM RIGHT TO LEFT.

*Exp.* IX. Rabbit. January 16, 1868. By the same process I wounded the brain.

The animal turned his head from the left side and inclined it slightly to the right. The eyes were both directed from the left side; the pupils were equal. In walking, the animal described a spiral movement from right to left in a curve of short radius.

January 16. The same symptoms.

January 17. Depression, pupils equal, the head and eyes are always directed to the left. The rabbit, when excited, described a spiral of short radius, from right to left.

He was killed by decapitation.

*Necropsy.* No sub-meningeal extravasation.

The instrument had wounded the right hemisphere very near the median portion of the interhemispheric fissure; it had turned obliquely to the left, and traversing the corpus callosum in the median line, had broken up the left optic thalamus. There was at this point a hæmorrhagic collection formed of cerebral substance mingled with blood, and embracing the entire thickness of the optic thalamus. The right optic thalamus was scarcely scratched.

The corpora striata were not injured.

I can compare with these nine experiments, IV and VI of the *Memoire sur le Ramollissement*, which my colleague M. J. Colard and I presented in 1865 to the Biological Society.

Dogs, the subjects of these experiments, presented, after the njection of water holding in suspension grains of tobacco, the phenomena of spiral rotation directed from the side opposite to a slight hemiplegia; and the necroscopic examination demonstrated to us in one case a focus of softening limited to the side toward which the spiral movement was directed, and in the other a focus of softening in each hemisphere, but more characteristic and more extensive, in surface and in depth, on the side toward which the spiral movement was directed.

*Rotary movements produced by unilateral lesions of the encephalic isthmus.*

As well among the inferior vertebrata as among the mammiferæ, the movements produced by lesions of the encephalic isthmus are sometimes a spiral, and sometimes a gyratory movement or rotation upon an axis.

I indicated previously one of the causes of obscurity relative to the determination of the direction of rotation upon an axis; I will not here revert to this subject, but I will simply recall that the direction of the movement upon an axis is the same as that of the spiral, that these two movements are performed in the direction indicated by the deviation of the eyes, and if the animal is displaced upon the ground into a direction opposite to the rotation which he effects upon his axis, that results only from the friction of the ground.

The direction of the rotation, in the case of unilateral lesion of the isthmus, is variable, according to the point of the isthmus which has been wounded, and it is not unfrequent to perceive, during the first moments which follow the operation, a spiral movement in a direction opposite to that which is established definitely some minutes later. This peculiarity was pointed out to me by M. Vulpian.

Let us examine the direction of this rotatory movement according to the different unilateral lesions of the isthmus.

Magendie,* specifying more clearly phenomena already perceived by Pourfour du Petit,† and by Serres,‡ observed that injury of the middle cerebellar peduncle occasioned rotary movements, produced always *from the same side as the section:* he remarked, morever, that the eye of the wounded side was directed downward and forward, that of the opposite side upward and backward. Magendie, by making a section of the cerebellum, in such a manner as to leave three-quarters to the left and one to the right uninjured, observed that the animal rolled to the right, and that the eyes were placed as if he had cut the right peduncle.

M. Longet,§ in his *Traité d'anatomie et de Physiologie du Système Nerveux*, asserts, as does M. Lafargue,‖ that rotation is effected, on the contrary, from the side opposite to the section of one of the middle cerebellar peduncles. Since that time, M. Longet ¶ has expressed the opinion that this difference between his results and those of M. Magendie resulted from the fact that the middle peduncle contains, especially posteriorly, non-decussating fibres, and decussating fibres anteriorly.

* Magendie, *Jour. de Phys. Expérim.*, 1824, t. IV., p. 399, et *Leçons sur les Fonctions et les Malad: du Syst. Nerv.* Paris, 1839.

† Pourfour du Petit, *Nouv. Syst. du Cerveau.* Paris, 1766.

‡ Serres, *Jour. de Physiol. Expérim. de Magendie.* 1823, t. III.

§ Longet, *Anat. et Physiol. du Système Nerveux.* Paris, 1842.

‖ Thèse de Paris, 1828.

¶ Voyez *Traité de Physiologie*, 1860, t. II, p. 406.

With the partial lesions of a cerebral peduncle, made immediately in front of the protuberance and a little beyond, M. Longet observed a spiral movement from the side opposite to the lesion, whilst Magendie, in cases of lesion of a cerebral peduncle, observed a circular movement from the side of the lesion.

M. Claude Bernard,* by wounding the cerebral peduncle behind the origin of the trigemini, observed that the revolution was made from the same side upon which the section had been performed. By making, on the contrary, the section in front of the origin of the trigemini, he observed the revolution from the side opposite to the section.

M. Schiff observed, in the case of lesion of the posterior parts of a cerebral peduncle, a rotary movement from the injured side.

M. Brown-Sequard,† in the case of lesion of some portion of the cerebral peduncle situated near the optic thalamus, observed a circular movement from the side opposite to the lesion; he observed rotation in the same direction with injuries involving the anterior and superior portions of the Pons Varolii, the portion of the medulla in which is inserted the glossopharyngeal, that portion of the spinal cord in which are inserted the first two or three pairs of nerves.

M. Brown-Sequard, on the contrary, observed a rotary movement from the injured side from injuries inflicted near the insertion of the inferior roots of the vagi nerve; he observed, with M. Martin Magron, the same direction of rotation following lesions of the medulla involving those portions in which are inserted the auditory and the facial nerve.

M. Vulpian,‡ after wounding the floor of the fourth ventricle, observed a rotation of the head and of the eyes from the side opposite to the injury, sometimes a spiral movement from the side indicated by the ocular deviation, sometimes a revolution upon an axis, in the same direction, and a displacement of the animal upon the ground in the opposite direction.

* *Mèmoire de la Soc. de Biologie.* Annèe, 1849.

† Voyez *Note sur les Mouvements Rotatoires dans Jour. de Physiologie* du Docteur Brown-Sequard, 1860, t. III. p. 724.

‡ Voyez *Effets des lesions du plancher du quatrième ventricule, etc.* (*Mèm. de la Soc. de Biologie.* Annèe, 1861.)

M. H. Paris published a case of hæmorrhage of the left lateral portion of the protuberance which occasioned in a cat a spiral movement from right to left, that is, from the side of the injury.

Messrs. Leven and Ollivier,* in numerous experiments performed upon the cerebellum, frequently observed the spiral or rotary movements occurring most frequently from the side opposite to the injury, but sometimes from the side of the injury.

It will be perceived, from this detail, that the directions of the spiral movement, and of the movements of rotation upon the axis produced by lateral lesions of the isthmus, may vary according to the parts involved.

In this case, as M. Vulpian remarks, in his *Cours de Physiologie*, (p. 586): "The direction of the movements of rotation spirally takes place most frequently from the injured side toward the healthy side, that is, from left to right if the injury is on the left. However, it is not rare to see the spiral movement performed in the inverse direction."

My personal experience exactly confirms these data.

Under this head should be classified lesions of the isthmus, lesions of one optic thalamus, and of one of the tubercula quadrigemina. Authors, as I have already stated, are not all agreed in this regard; but it is very possible that in attacking posteriorly the optic thalamus, the lateral portions of the isthmus are excited; the effects produced are then due to the encephalic isthmus. It has been apparent that, in my experiments, I have always obtained, by attacking the optic thalamus from above downward, a rotation from the side of the lesion.

As to the tubercula quadrigemina or bigemina, I have convinced myself by experiments made upon frogs, in which these parts are readily discovered, that the direction of the rotation varies according to the part injured. Rotation seemed to me to be made most habitually in the direction of the injury, if that were situated entirely in the anterior portion of the tubercle, and from

* M. Leven and A. Ollivier *Recherches sur la Phys. et la Path. du Cervelet*, (*Arch. Gén. de Méd.*, 1862, t. II.). Voyez aussi Gratiolet et Leven, *sur les Mouvements de Rotation sur l'axe que determinent les Lesions du Cervelet*, (*Compte Rendu, Ac. Sc.*, 1860). Leven, *Nouv. Rech. sur la Phys. et la Path. du Cervelet*, (*Mém. Soc. de Biolog.*, 1864.)

the opposite side if the middle or posterior portion of the tubercula bigemina were attacked. In this last case the rotation was most marked and most persistent.

These differences in the results obtained after lesions of the isthmus, may be readily understood when it is remembered that the decussation of nervous fibres is not so complete at this as at a higher level.

The preceding experiments were made in view of my inaugural thesis,* in which I published them, comparing them to a very frequent but little known symptom of apoplexy of cerebral origin, toward which M. Vulpian directed my attention, I mean the conjugate deviation of the eyes and rotation of the head, from the side opposite to the hemiplegia.

M. Vulpian, in a course upon the physiology of the nervous system, delivered, in 1864, at the Museum of Natural History, compared this phenomenon to the movements of rotation observed in animals, and the following year I published in the *Gazette Hebdomadaire* a short note upon this point.

I have been able to collect fifty-eight observations, of which fifty-five were followed by necropsy, the majority being due to the kindness of Messrs. Vulpian and Charcot.

After studying these observations, and comparing them with the results of my experiments relative to the rotary movements observed in animals subsequent to unilateral cerebral lesions, I have succeeded in demonstrating the identity of these phenomena.

It was apparent indeed from the experiments reported above, that rotation resulting from lesion of a cerebral hemisphere was accompanied by conjugate deviation of the eyes and by rotation of the head from the side of the wounded hemisphere. When the injury was seated in the encephalic isthmus, the direction of the rotation was subject to variation, but was effected in the direction indicated by the ocular deviation.

In all the observations which I have been able to gather, of rotation of the head and eyes accompanying a lesion of the brain properly speaking, this deviation of the eyes and of the head took

* *De la Déviation Conjuguée des Yeux, et de la Rotation de la Tête dans certains cas de Hémiplégie.* Thèses de Paris, 1868, chez V. Masson et fils.

place from the side of the wounded hemisphere, from the side opposite to the hemiplegia.

I have cited cases in which this deviation occurred after even superficial lesions of the brain, but the most numerous cases were due to more profound injuries, approximating the corpus striatum and the peduncular irradiation. In four observations, on the contrary, the encephalic injury was situated in the encephalic isthmus, in which I include the cerebellum; rotation of the head and eyes occurred three times from the side opposite to the injury, and once from the side of the injury.

It should be added, that in several of these observations a tendency was observed, to a movement of translation from the side indicated by the ocular rotation, which approximates these phenomena still more closely.

I will no longer dwell upon the facts developed in my inaugural thesis, as it is my intention to treat here only the purely physiological portion of the treatise. W. H.

